# Body Image Perception in Adolescents: The Role of Sports Practice and Sex

**DOI:** 10.3390/ijerph192215119

**Published:** 2022-11-16

**Authors:** Stefania Toselli, Natascia Rinaldo, Mario Mauro, Alessia Grigoletto, Luciana Zaccagni

**Affiliations:** 1 Department of Life Quality Studies, University of Bologna, 47921 Rimini, Italy; 2 Department of Neuroscience and Rehabilitation, Faculty of Medicine, Pharmacy and Prevention, University of Ferrara, 44122 Ferrara, Italy; 3 Department of Biomedical and Neuromotor Sciences, University of Bologna, 40126 Bologna, Italy

**Keywords:** adolescence, dissatisfaction, sport participation, volleyball

## Abstract

Concerns about weight and body image are common among adolescents, as adolescence represents a time of intense and rapid change. This cross-sectional study assessed the difference in body image perception by sex, weight status and sports practice in a sample of Italians aged 13–18 years. For this purpose, we considered a sample of 140 adolescents subdivided into two groups: a group of sports-playing teenagers practicing volleyball (39 males and 26 females), and a group of teenagers not actively involved in sports (24 males and 51 females). Body mass index (BMI), body image variables, and sports practice were examined. Due to COVID-19 limitations, height and weight were self-reported and BMI was calculated as weight (in kilograms) divided by the square of height (in meters). Body image perception was assessed by the short version of the Body Shape Questionnaire (BSQ-14) and by the Stunkard Body Silhouette Chart. Two-way ANCOVAs adjusted for age were performed to test the differences between sexes and sports groups. Adolescent volleyball players had a lower incidence of weight disorders. Weight status significantly differed between those who played sports and those who did not, but there was no significant difference in weight status between sexes. On the whole, sports players and males showed higher results than non-sports players and females. The highest level of body image dissatisfaction was found in non-sports-playing females, while sports-playing males showed the lowest. The present study confirms the positive link between sport and body image and can be of support to lead adolescents to adopt a healthier and more active lifestyle. Further research is needed to validate our findings through a longitudinal study during the entire period of adolescence. In addition, it would be interesting to validate the results on a larger sample, also taking into account socio-demographic variables and including athletes engaged in different types of sports.

## 1. Introduction

Body image refers to a multidimensional construct that involves perceptions and attitudes towards oneself that are related to one’s body, including thoughts, beliefs, feelings, and behaviors. It usually incorporates body size estimation, body attractiveness, and emotions associated with body shape and size [1]. There are two aspects connected with body image: weight misperception (a perceptual aspect of body image relating to over- or under-estimates of weight), and body dissatisfaction. These are two separate constructs since one can be quite accurate in the perception of one’s body size and shape, and yet still be dissatisfied with it [2,3,4]. Body image can in fact be defined as the self-perception of the physical self, and the feelings and thoughts that arise from that perception [5]. Disorder in any of these domains is referred to as concern with body image or negative body image.

A period of particular vulnerability toward body image perception is represented by adolescence, due to the physiological, social, and psychological changes that adolescents go through [4,6,7]. To accommodate the different phases of development in the second decade of life, adolescence is often divided into early (10–13 years), middle (14–16 years), and late (17–19 years). All the above-mentioned changes become more pronounced by late adolescence and can lead to greater concern over one’s physical appearance [7].

Among adolescents, the frequent search for physical characteristics outside of reality can determine dissatisfaction with one’s body image [3,8]. In addition, in the society of today, there is an idealization of a perfect body, which, especially in adolescents, if not achieved, can lead to body image disturbance, often related to negative health and behavior effects. Having a body image close to the socially accepted ideal of beauty is conceptually associated with possessing other positive characteristics such as being successful or healthy [5,9]. Studies have demonstrated that adolescents’ body image concerns are associated with disturbed eating attitudes and behaviors that might be the precursor of clinical eating disorders [10].

Several biological factors influence the risk of body image disturbance during adolescence: age, sex, puberty, and body characteristics, and each of these merits attention [11]. A perception of being too fat is the most substantial predictor of a wide range of problems, and it can affect young people whether they are of normal weight or overweight [5]. Moreover, concern over body image differs according to sex: male teenagers generally wish to have a more muscular body, while the female ideal of beauty coincides with thinness, in most cases below a healthy size [4,5]. Especially in girls, the perception of being overweight and dissatisfaction with body size appear to lead to dieting and weight control behaviors [1,12]. Although a higher proportion of girls fall into the normal weight category compared to boys, girls tend to have a greater misperception about their body image, and this can lead to more weight-control behaviors. BMI and weight status are connected both with physical activity (PA) and body image perception. The association between BMI and physical activity is likely to be bi-directional: higher levels of physical activity prevent gains in adiposity, while high body weight can lead to lower physical activity levels [13]. BMI is one of the most important factors influencing body image and body satisfaction. As a biological component, BMI has been found to contribute to body image and fear of negative evaluation (fear that one will be evaluated unfavorably because of one’s appearance). Overweight individuals are more likely to report a sense of fear associated with being negatively evaluated while engaging in social situations, compare to their normal-weight counterparts [14]. Teenagers with higher BMI may feel discriminated against, due to their image, which can lead them to present internalizing symptoms in the long term. This is related to an increased awareness and internalization of social attitudes around weight throughout childhood, which is manifested in psychological problems in adolescents who are unhappy with their body image [5].

Some studies have demonstrated that sports participation and physical activity (PA) practice can influence body image in adolescents [15,16,17,18,19]. PA and participation in sports are highly promoted for adolescents because of the numerous benefits for health outcomes, positive cognitive function, and academic outcomes, leading to a lower risk of experiencing depression [20,21]. Tebar et al. reported that PA is favorably associated with lower adiposity: they found that decreases in PA levels from childhood to adolescence can be related to an increase in body fat in both sexes, while increases in PA levels were associated with a decrease in body fat [22]. Many of the benefits were observed with an average of 60 min of moderate-to-vigorous physical activity (MVPA) daily, although PA beyond 60 min of MVPA daily appeared to boost health outcomes further. Lower body dissatisfaction was observed in adolescents of both sexes who play sports: sports practice can contribute to lower adiposity levels and, consequently, to lower body dissatisfaction in the adolescent population [23,24,25]. Liu et al. found that PA in itself is associated with self-concept and self-esteem in children and adolescents [26]. However, results of studies exploring the associations of body image and sports practice in adolescents are not consistent, especially according to sex. Tebar et al. observed that sports practice from infancy to adolescence was associated with lower body dissatisfaction in boys, but not in girls [22]. These gender differences have deleterious consequences, such as the abandonment of sports by girls, leading to negative effects at a physical and psychological level. Boys and girls socialize differently, through gender-stereotyped beliefs and behaviors in the family, physical education classes, and sports contexts where girls and boys are not encouraged in the same way [27,28]. In Italy, gender disparities in sports participation have been observed in girls aged 11–19 (41% of girls vs. 59% of boys) [27]. Despite the importance attributed to sports practice, the experience of adolescents in this regard has not been widely investigated, and studies of body image concerns in adolescents involved in sports are rare and inconclusive.

Therefore, the present study aimed to expand knowledge of this issue, assessing weight status and body image perception in a sample of adolescents of both sexes who practice volleyball, and comparing the results with those of adolescents who do not engage in physical sporting activity. We expected that adolescents participating in sports would demonstrate a lower prevalence of being overweight or obese, and a better body image perception, together with lower levels of dissatisfaction with respect to sex-matched controls. We also aimed to verify whether gender moderated the association between body image and sports practice.

## 2. Materials and Methods

### 2.1. Participants

A cross-sectional study was carried out between December 2020 and March 2021 on a sample of 140 Italian adolescents from northern Italy, subdivided into two groups: a group of sportive teenagers and a group of non-sportive teenagers. The former group was composed of 65 young volleyball players: 39 males (mean age: 15.6 ± 1.1 years) practicing volleyball at the Treviso Volley sports club, and 26 females (mean age: 16.2 ± 1.2 years) practicing volleyball at the Albatros Volleyball sports club, in Treviso. All subjects practiced volleyball for 10 h/week. The choice of volleyball was dictated by the fact that this sport is widely practiced by both sexes. Inclusion criteria were age range between 13 and 18 years, practicing volleyball, and being Italian. The non-sportive group was composed of 75 high-school students who did not practice any sports: 24 males (Mean age: 16.2 ± 1.1 years) and 51 females (Mean age: 16.0 ± 1.2 years). The non-sportive sample was selected based on the absence of sports participation from the 212 subjects (163 females and 49 males) of the school (located in northern Italy; total 798 students) who voluntarily participated in the survey (26.6% percentage of adhesion). Inclusion criteria were age range between 13 and 18 years, not practicing any sport, and being Italian. All the subjects voluntarily participated in the study, giving their verbal assent. Written informed consent was provided by parents/tutors before the study began. The study was approved by the Bioethics Committee of the University of Bologna (approval code: 25027).

### 2.2. Procedures

#### 2.2.1. Anthropometric Traits

Since the study was carried out during the COVID-19 public health emergency period, in which external personnel were not able to access Italian school facilities or gyms, anthropometric characteristics (height and weight) were self-reported by each participant and BMI was calculated as weight (in kilograms) divided by the square of height (in meters). This index was used to assess the weight status: each subject was thus classified into underweight, normal weight, overweight or obese, according to the Cole cut-off values by sex and age [29,30].

#### 2.2.2. Body Image Perception

To assess body image perception, we used two approaches: a questionnaire and figural stimuli. We used the short version of the Body Shape Questionnaire (BSQ-14) [31], translated into Italian and validated by Matera et al. (2013) [32]. The internal reliability (Cronbach’s alpha) of this questionnaire was 0.93 [25]. This assesses body dissatisfaction and treatment of eating disorders; it includes 14 items with a six-point Likert scale ranging from never (1 point) to always (6 points). The total score was calculated on the sum of all the values multiplied by 34/14 and subsequently related to the specific thresholds that refer to the complete form of the questionnaire: a score below 80 indicates “no concern”, between 80 and 110 “slight concern”, between 111 and 140 “moderate concern”, and a score above 140 indicates “marked concern”.

In the second approach, body image perception was assessed using the Stunkard Body Silhouette Chart [33]. The subjects were shown nine male or female silhouettes, ordered in morphology from emaciation to obesity, and were asked to select the silhouette which they believed was most similar to their own figure (‘feel figure’) and the silhouette which they considered the ideal body (‘ideal figure’). The discrepancy between the feel figure and the ideal figure represents the degree of body image dissatisfaction (FID or Feel minus Ideal Discrepancy) [34]. The FID index was calculated by subtracting the score of the figure selected by adolescents as the ideal figure from the one selected as their feel figure. A positive FID score indicated a desire to be slimmer, while a negative score indicated a desire to be larger. A FID score of 0 indicated no discrepancy and thus no dissatisfaction (same figure chosen as feel figure and as ideal).

Incorrect perception of weight status was evaluated by means of FAI (Feel weight status minus Actual weight status Inconsistency) [35]. We calculated the FAI index by subtracting the conventional code assigned to the actual weight status of the participant, according to the BMI assessed by Cole cut-off values by sex and age [29,30], (1 = underweight; 2 = normal weight; 3 = overweight; 4 = obese), from the code of her/his perceived weight status (1 = underweight; 2 = normal weight; 3 = overweight; 4 = obese), obtained using the classification recommended by Bulik et al. [30], which assigns a specific weight status category to each silhouette. According to this classification, silhouettes 1, 2 and 3 correspond to underweight (=1), silhouettes 4 and 5 correspond to normal weight (=2), silhouettes 6 and 7 correspond to overweight (=3), silhouettes 8 and 9 correspond to obesity (=4). A FAI score of zero indicates no inconsistency in weight status perception, a positive score indicates that feel weight status is overestimated, and a negative score indicates that feel weight status is underestimated. Two more questions were asked. The first question, named ‘attractive figure’, was: “In your opinion, what is the ideal figure according to the other sex?” The second question, named ‘other attractive figure’, involved showing the silhouettes of the opposite sex, and asking: “What is your ideal figure among the silhouettes of the other sex?”

In addition, we calculated the FAD index (Feel minus Attractive Discrepancy), to evaluate the discrepancy between the feel silhouette and the attractive silhouette [36]. A FAD score of 0 indicates a perfect matchbetween the feel silhouette and the attractive silhouette for the opposite sex. When the score is different from 0, the level of discrepancy reflects the degree of dissatisfaction. A positive FAD score indicates that the feel silhouette is bigger than the attractive silhouette for the opposite sex; a negative FAD score indicates that the feel silhouette is smaller than the attractive silhouette for the opposite sex.

### 2.3. Statistical Analysis

To identify the minimum recommended sample size for the study, we assessed an ‘a priori: computer required sample size given α, power and effect size’ by G*Power (3.1.9.4, Universität Kiel, Kiel, Germany). An ANCOVA was selected as the *F* test of all the test family, imputing the following parameters: α = 0.05; (1 − β) = 0.80; effect size f = 0.25; numerator df = 1; number of groups = 4; number of covariates = 1. The outcome parameters thus calculated detected a sample size of 128 participants, but additional subjects were involved to ensure availability of data in case of problems with data collection. Variables normality was verified with the Kolmogorov-Smirnov test. Descriptive statistics (means and SD) were calculated. Percentage frequency was determined for qualitative variables (weight status, weight control, and concern about body image), and differences in the frequencies were tested by the chi-squared test. Bivariate correlation was carried out to value the relationships between the two methods of assessment of body image dissatisfaction and between BMI and FID. Two-way ANCOVAs adjusted for age were performed on the items of the BSQ-14, anthropometric traits, and body image variables to test the differences between sexes and sportive groups. Partial ETA squared was computed to obtain the effect size. Multiple linear regression analyses were performed to assess the association between the sports group and sex (independent categorical variables) on FID (dependent variable). The model was adjusted for age and BMI, inserted as continuous independent variables. Multicollinearity was evaluated by a variance inflation factor (VIF): a value of VIF > 10 indicated excessive multicollinearity. The significance level for all statistical tests was set at *p* < 0.05. The data analysis was performed using Statistica for Windows version 8.0 (Stat Soft Italia SRL, Vigonza, Padua, Italy) and MedCalc software version 14 (MedCalc Software, Ostend, Belgium).

## 3. Results

An initial observation regarding the sample relates to the gender ratio of the teenagers who decided to participate in the study: 77% of the respondents were female. Among these, the students selected on the basis of the absence of sport practice were 31% of the females in the sample, and 49% of the males.

Sports-playing participants of both sexes were taller and with a significantly lower BMI than their non-sports-playing counterparts. No differences among groups were found when weight was compared. In the comparison between sexes, males were significantly taller and heavier than females, but the mean BMI did not show significant differences. Moreover, the interaction between sports practice groups and sex did not show as significant in any of the anthropometric characteristics (Table 1).

As regards the weight status (Table 2), non-sports-playing participants of both sexes showed a lower percentage of normal weight subjects in comparison with their sports-playing peers, and a higher percentage of being overweight and obesity.

In the sports-playing group, there were no obese subjects, while about 18% of males and 8% of females fell into the overweight category. The comparison between sportive and non-sportive groups showed statistically significant results, but males and females had similar BMI category distribution. The interaction between sex and groups showed significant differences, with a higher percentage of subjects in the extreme categories in the non-sportive male group.

With respect to BSQ-14 (Table 3 and in the Supplementary Material Table S1), both the practice of sport and sex influenced the results regarding the items, with sportive and male groups showing better results than non-sportive and female groups.

Analyzing in detail the individual results, the score of the females was always significantly higher in comparison to males, demonstrating greater concern about their shape. Considering the differences between sportive and non-sportive groups, just some items resulted in significant differences between the groups, with higher concern always in the non-sportive group. In particular, the sportive and non-sportive groups had similar concerns in that being with thin people made them feel uncomfortable about their own body shape, that concern about their shape made them go on a diet, that it was not right that other people were thinner than themselves, that eating sweets, cakes or other high-calorie foods made them fat (Supplementary Material, Table S1).

Considering the specific thresholds of the total score, males of both groups showed a higher percentage of no concern (Table 3, second part) and this is particularly evident in the sportive group that showed over 70% of no concern and 0% of marked concern.

Within the group of males, a higher percentage of marked, moderate and slight concern can be found in the non-sportive groups. These results are consistent in the group of females in which the percentages of moderate and marked concern were also higher in the non-sportive group. Regarding the mean level of concern, a significant difference was observed among sportive and non- sportive groups and sexes, with higher concern in non-sportive subjects and especially in females. In all the items and the total level of concern, the interaction between sports groups and sex was non-significant, demonstrating that the minor concern in sportive subjects did not depend on sex.

Body image perception showed significant differences between groups as regards the feel figure (Table 4): in accordance with the different weight status, sportive participants chose thinner silhouettes than their non-sportive peers. Sportive females showed a significantly slimmer ideal figure in comparison to their non-sportive counterparts, while no significant differences were exhibited in the groups of males. As regards FID, no significant differences were shown among sexes; however, the comparison between sportive and non-sportive groups revealed that the latter were more dissatisfied with their body size. The highest level of dissatisfaction was found in non-sportive females and the lowest level in sportive males.

The results of the correlation between BMI value and FID index revealed a significant positive correlation in the total group (r = 0.45; *p* < 0.001) and both in males (r = 0.39; *p* = 0.002) and females (r = 0.52; *p* < 0.001), indicating that dissatisfaction increases with the increase in BMI. Moreover, the correlation between the two variables of body image dissatisfaction (total score by BSQ-14 and FID) was significant and positive both in male groups (r = 0.48; *p* < 0.001) and in female groups (r = 0.49; *p* < 0.001).

As regards the consistency of the perception of weight status detected by FAI, non- sports-playing students tended to underestimate their weight status, while sports players tended to overestimate it, and the mean values of the four groups were close to zero.

All groups showed a positive FAD score, indicating that the feel silhouette always scored higher than the attractive silhouette for the opposite sex. The comparison between sports groups was statistically significant, with lower discrepancy among the sports-playing groups. Females thought that the attractive body image preferred by the other sex was thinner than that chosen by males, and the comparison was almost significant.

In comparison with the non-sports-playing group, sports players chose thinner silhouettes as the ideal of the opposite sex, but the comparison did not reveal significant results.

The need to control their weight and the desire to lose it were more pronounced in non-sports-players and in females than in males (Table 5).

Significant differences were observed between sports groups, sexes, and interaction between both the conditions for the desire to lose weight, and between sports-players and non-sports-players in both sexes to keep track of their own weight.

Table 6 reported the results of the multiple linear regression model with FID as the dependent variable. Adjusting for age and BMI, the variables “sex” and “sports group” showed results significantly associated with body image satisfaction, with an explained variance of 31%. In particular, FID was higher in the female group and lower in the sports-playing group. Moreover, for every 1 kg/m^2^ increase in BMI, FID rose by 0.17 points (B parameter, not included in the table).

## 4. Discussion

The present study aimed to assess weight status and body image perception in a sample of sportive and non-sportive adolescents, also taking into account gender differences.

A first observation regards the non-sportive sample should be considered. We selected that group from high-school students who did not practice any sports, and two aspects emerged: overall, most of the participants who decided to participate in the study were females, and, unexpectedly, among all the students in the school, girls played more sports than males. Generally, the prevalence of people who play sports is lower among girls compared to boys: globally, 77.6% of boys and 84.7% of girls aged 11–17 years are insufficiently physically active [37], and within the EU, the prevalence of physical inactivity is 64.3% and 73.8%, respectively [38].

Regarding the relationship between BMI and sport practice, our results suggest that adolescent volleyball players have a lower BMI and consequently a lower incidence of weight disorders. Significant differences in weight status were observed between sports-players and non-sports-players, but not between sexes. Even if in the literature the relationship between sports participation during childhood and adolescence and indicators of overweight/obesity, such as BMI, is inconclusive [39,40], in the present study, sport seems to be an important protective factor against overweight/obesity risk. This is in accordance with other studies on this topic [41,42]; in particular, Drake et al. reported that compared with other forms of PA, sports participation had the strongest and most consistent inverse relationship with elevated weight status [42]. This aspect is particularly important in adolescence, in which, especially in females, age-related transitions can lead to an increase in body fat. Obese adolescents may be less likely to take part in PA because of fear of poor performance and stigmatization [43]. The prevalence of obesity increases rapidly during adolescence and young adulthood, and its causes are complex and multifactorial, but evidence suggests that prevention is critical, as overweight/obese adolescents are unlikely to improve their weight status as they progress into young adulthood [13]. PA and sedentary behaviour are considered cornerstones for preventing unhealthy weight gain, with extensive review-level evidence available [21].

Weight status is confirmed to be an important factor in influencing body image perceptions among teenagers. In fact, adolescence is strongly connected to body image dissatisfaction: specifically, research consistently shows that greater body mass index (BMI) is associated with heightened weight concerns among both adolescent girls and boys [44,45]. The changes that occur during adolescence are among the most intense during growth and include changes in weight, height, body shape, and body composition, as well as primary and secondary sexual characteristics. It is important to point out that these physical changes also coincide with heightened exposure to cultural ideals of beauty. For girls, cultural expectations emphasize being thin, and the changes associated with puberty, such as the increase in adiposity and hip enlargement, can be felt negatively and seen as inconsistent with the prototype and societally valued thin ideal. Weight status significantly affects the way girls perceive their body, and girls with a higher body weight have a more critical attitude toward their appearance. Especially in girls, the perception of being overweight and dissatisfaction with body size appear to lead to deleterious consequences, such as dieting and weight control behaviors [1,12]. Body dissatisfaction is in fact connected prospectively to unhealthy weight-control behaviors, binge eating, and lower levels of physical activity [6]. Although adolescent boys have a better perception of their image, they are not immune to body image problems during adolescent development. In particular, the increase in height and muscle mass associated with puberty brings some boys closer to cultural expectations of being tall and muscular. Body dissatisfaction among males is understudied and needs more and specific attention. Generally, some adolescent boys want to be thinner whereas others desire to be bigger (i.e., more muscular), thus boys tend to be more dissatisfied when their body mass index (BMI) is below or above average [45,46]. According to some authors [46,47], despite females having higher body dissatisfaction scores than boys, the strength of the associations between body dissatisfaction and impairment does not significantly differ between the sexes.

To assess concerns about body shape and size, two methods were used: BSQ-14 and body image perception by silhouettes. As regards the BSQ-14 test, both the practice of sport and gender influenced the answers and, on the whole, sports players and males showed better results than non-sports-players and females, respectively. Some issues related to thinness were common among females, presenting no differences between the sportive and non-sportive females. Some issues connected with thinness and diet (such as a comparison with thin people, or the thought that it is unfair that other people are thinner than themselves, or following a diet because of concern over their body image) seem to be typical of females, regardless of whether they practice sports. Similarly, males of both groups shared common concerns about issues related to the comparison of their own body shape with that of others. These aspects suggest common socio-cultural pressures in body image related to gender.

The influence of sport emerged with more emphasis on some points, which differentiated sports players and non-sports-players across both gender groups. Non-sportive females showed the greatest discomfort concerning issues related to the judgment of other people. Undressing, feelings of shame towards their own body, poor acceptance of the reflection of their image in the mirror, and being seen to have rolls of fat around their waist or stomach gave cause for concern. Questioned on the same issues, sportive males showed the best results, while sportive females and non-sportive males presented intermediate values and did not differ from each other. In this regard, sport seems to be a protective factor, regardless of sex.

As a whole, considering the threshold of concern, males of both groups presented a tendency to show less concern than females, and this is particularly evident in sports-playing groups.

Body image perception by silhouettes confirmed the differences connected to sport and sex. The highest level of body image dissatisfaction (FID) was found in non-sportive females, while sportive males showed the lowest. The consistency of self-perception of weight status, detected by the FAI index, was also connected to sports practice: non-sports players tended to underestimate their weight status, while sports players tended to overestimate it.

Differences based on sports practice were also found in the opinion related to the choice of the attractive silhouette and other attractive silhouette: sports players of both sexes chose thinner silhouettes than their non-sportive peers, thus showing different ideals.

The need to control weight and the desire to lose it were more pronounced in non-sportive participants than in those who played sport. The desire to lose weight differed between females and males, being generally more noticeable in females.

The present study adds details about adolescence, a critical age for PA and sports practice. During adolescence, generally, PA levels and sports practice decrease while sedentary time increases more than at other ages, the changes being more evident in girls than in boys [48,49].

Our findings are in accordance with previous studies, which reported that adolescent girls showed higher levels of body dissatisfaction than their male counterparts [14,40]. Moreover, it should be noted that in the present study, people who do not practice sports showed a greater dissatisfaction and uneasiness with their body compared to those who do play sports. This finding extends the literature supporting lower levels of body dissatisfaction and higher levels of body esteem in people who play sports, compared to those who do not [50,51]. According to Soulliard et al., sport provides the opportunity for individuals to form a close and appreciative relationship with their bodies, given the need for increased body awareness and attentiveness [16]. Sportive males reported higher levels of body satisfaction compared to sportive females, so, although sports practice is a positive factor in the development of a positive body image, sociocultural pressures related to body image probably still have differing impacts on female and male athletes.

The limitations of this study relate primarily to the fact that the research design is cross-sectional, limiting causal inferences. In addition, the self-reported measurements of height and weight carried out in this study due to COVID-19 may have influenced the accuracy of the results. We have no information regarding sociodemographic variables, so we could not assess the influence of this aspect in explaining some of the links between sports participation and body perceptions. This study only evaluated volleyball players, and other sporting groups were not considered. Future research may validate our findings through a longitudinal study during the entire adolescence period. Furthermore, the sample size was limited. Further research on larger samples is needed to test the validity of our findings, including adolescents engaged in sports other than volleyball.

## 5. Conclusions

The results of our study demonstrated that youngsters who practice regular sports activities have a healthier weight status and body image perception and less dissatisfaction than their non-sportive peers. Moreover, although females showed greater dissatisfaction and concern with their external appearance compared to their male counterparts, non-sportive boys were nonetheless also affected by body image concerns during adolescence. Thus, the present study confirms the positive link between sport and body image and can be of support to lead adolescents to abandon sedentary lifestyles and adopt healthier active ones, and to promote better awareness of their body. Actions to promote and raise awareness of participation in sport should be increased and addressed both to young people and to the adults around them (e.g., parents, educators, and teachers). In addition, especially in the school context, it is important to make educators and teachers more sensitive to the value of correct body-image perceptions, so that they can pay attention to this issue and support students, while also promoting healthy lifestyles and positive body image in adolescents. Assessing body-image issues may be important when identifying adolescents at risk.

## Figures and Tables

**Table 1:** Anthropometric characteristics of adolescents according to sport group and sex.

	No Sport M		Sport M		No Sport F		Sport F		Group		Sex		Group*Sex	
Variable	M	SD	M	SD	M	SD	M	SD	F	*p*	F	*p*	F	*p*
Height (cm)	175.2	8.0	182.0	8.3	162.8	6.5	172.2	7.5	54.97	0.000	81.35	0.000	0.05	0.826
Weight (kg)	73.9	18.8	70.6	10.3	59.2	11.8	63.5	8.8	1.98	0.162	27.93	0.001	1.28	0.259
BMI (kg/m^2^)	23.9	5.1	21.2	2.1	22.3	4.0	21.3	2.0	4.45	0.037	1.76	0.187	1.15	0.286

* interaction between group and sex.

**Table 2:** Distribution (%) of weight status of adolescents according to sport group and sex.

	No Sport M	Sport M	No Sport F	Sport F	Group *p*	Sex *p*	Group*Sex *p*
Weight Status					0.001	0.845	0.008
Underweight	16.7	2.6	9.8	3.9			
Normal weight	37.5	74.5	64.7	88.5			
Overweight	33.3	18.0	21.6	7.7			
Obese	12.5	0.0	3.9	0.0			

* interaction between group and sex.

**Table 3:** Total final score of BSQ and distribution (%) of adolescents divided in concern category by sport group and sex.

	No Sport M	Sport M	No Sport F	Sport F	Group			Sex			Group*Sex		
	Mean (SD)	Mean (SD)	Mean (SD)	Mean (SD)	F	*p*	Partial ETA^2^	F	*p*	Partial ETA^2^	F	*p*	Partial ETA^2^
Total score	80.14 (32.97)	71.86 (19.45)	101.19 (35.29)	88.74 (25.61)	6.6	0.011	0.47	14.3	0.000	0.96	0.0	0.847	0.000
Category	%	%	%	%		0.011			0.002			0.005	
no concern	54.2	71.8	33.3	30.8									
slight	25.0	23.1	25.5	50.0									
moderate	12.5	5.1	19.6	7.7									
marked	8.3	0.00	21.6	11.5									

* interaction between group and sex.

**Table 4:** Body image perception of participants according to sport group and sex.

	No Sport M		Sport M		No Sport F		Sport F		Group			Sex			Group*Sex		
Variable	m	SD	m	SD	m	SD	m	SD	F	*p*	Partial ETA^2^	F	*p*	Partial ETA^2^	F	*p*	Partial ETA^2^
Feel figure	4.46	1.82	3.41	0.68	4.00	1.56	3.27	1.00	10.7	0.001	0.073	1.7	0.197	0.012	0.3	0.576	0.020
Ideal figure	3.58	0.97	3.36	0.67	3.04	0.92	2.65	0.63	0.3	0.602	0.002	22.7	0.001	0.144	1.8	0.180	0.013
Attractive figure	3.54	1.35	3.21	0.70	3.27	0.83	3.00	0.89	2.9	0.630	0.002	0.2	0.089	0.021	0.2	0.632	0.002
OAF	3.63	0.97	3.18	0.68	3.39	0.96	3.31	0.55	0.0	0.885	0.000	0.4	0.524	0.003	0.2	0.659	0.001
FID (score)	0.88	1.36	0.05	0.83	0.96	1.50	0.62	0.85	10.9	0.001	0.075	2.7	0.100	0.020	2.3	0.135	0.016
FAI (score)	−0.08	0.58	0.03	0.43	−0.02	0.65	0.15	0.37	3.3	0.070	0.024	0.1	0.760	0.001	0.6	0.423	0.005
FAD (score)	0.92	2.02	0.21	0.83	0.73	1.51	0.27	1.15	8.0	0.006	0.056	0.0	0.881	0.000	0.7	0.401	0.005

Note: OAF = other attractive figure; FID = feel minus ideal discrepancy; FAI = Feel weight status minus Actual weight status Inconsistency; FAD = Feel minus Attractive Discrepancy. * interaction between group and sex.

**Table 5:** Opinion of participants regarding weight control according to sport group and sex.

	No Sport M	Sport M	No Sport F	Sport F	Group *p*	Sex *p*	Group*Sex *p*
Have you ever tried to lose weight?					0.001	0.040	0.006
I don’t need it	25.0	30.8	13.7	11.5			
Never	20.8	23.1	11.8	23.1			
Sometimes	37.5	43.6	41.2	65.4			
Often	12.5	2.6	23.5				
All time	4.2		9.8				
Do you keep track of your weight?					0.018	0.436	0.077
I don’t need it	12.5	10.3	9.8	0.0			
Never	25.0	15.4	15.7	3.8			
Sometimes	41.7	64.1	51.0	84.6			
Often	8.3	2.6	15.7	0.0			
All time	12.5	7.7	7.8	11.5			

* interaction between group and sex.

**Table 6:** Multiple linear regression model that analyzes the role of sex and sport as predictors of FID (dependent variable), adjusting for age and BMI.

Variables	β	t	*p* Value	VIF
Age (years)	−0.229	−2.927	0.004	1.2
BMI (kg/m^2^)	0.497	6.757	0.000	1.1
Sex (Female)	0.194	2.615	0.010	1.1
Group (sportive)	−0.193	−2.394	0.018	1.3
*p* value	0.000			
R^2^	0.329			
Adjusted R^2^	0.309			

## Data Availability

Data is available upon request.

## Supplementary Materials

The following supporting information can be downloaded at: https://www.mdpi.com/article/10.3390/ijerph192215119/s1, Table S1: BSQ-14 items of the participants according to sport group and sex.
