# Supplementary material for: Body Image Perception in Adolescents: The Role of Sports Practice and Sex
Source: Int J Environ Res Public Health. 2022 Nov 16;19(22):15119. doi: 10.3390/ijerph192215119 (PMC9690021; doi:10.3390/ijerph192215119)
Supplement: Supplementary file 1 [file ijerph-19-15119-s001.zip › ijerph-2007966-supplementary.pdf]

Table S1. BSQ-14 items of the participants according to sport group and sex

	No sport M		Sport M		No sport F		Sport F		group			sex			group*sex		
Item	M	SD	M	SD	M	SD	M	SD	F	p	Partial ETA^2	F	p	Partial ETA^2	F	p	Partial ETA^2
Have you been so worried about your shape that you have been feeling you ought to diet?	2.25	1.26	1.82	1.17	3.43	1.84	2.77	1.42	3.2	0.075	0.023	15.7	0.000	0.102	0.2	0.640	0.002
Has being with thin people made you feel self-conscious about your shape?	4.58	1.91	5.05	1.21	3.88	1.85	4.00	1.39	2.0	0.160	0.015	9.7	0.002	0.067	0.7	0.400	0.005
Have you noticed the shape of other people and felt that your own shape compared unfavourably?	2.29	1.55	2.33	1.06	3.90	1.68	3.42	1.14	1.1	0.289	0.008	29.4	0.000	0.179	0.8	0.379	0.006
Has being undressed, such as when taking a bath, made you feel fat?	1.92	1.50	1.36	0.67	3.02	1.94	2.12	1.24	9.3	0.003	0.065	13.2	0.000	0.069	0.1	0.691	0.001
Has eating sweets, cakes or other high calorie food made you fell fat?	2.29	1.57	2.00	1.36	2.88	1.73	2.77	1.50	10.0	0.324	0.007	6.2	0.014	0.044	0.2	0.632	0.002
Have you felt excessively large and rounded?	2.46	1.61	1.62	0.88	3.16	1.80	2.38	1.24	10.9	0.001	0.075	8.4	0.004	0.059	0.1	0.718	0.001
Have you felt ashamed of your body?	1.67	1.61	1.13	0.47	2.92	1.99	1.69	1.23	11.2	0.001	0.079	12.2	0.001	0.083	1.2	0.272	0.009
Has worry about your shape made you diet?	2.08	1.41	1.77	1.09	2.75	1.62	2.65	1.38	0.7	0.412	0.005	9.6	0.002	0.066	0.2	0.634	0.002
Have you thought that you are in the shape you are because you lack self-control?	2.21	1.72	1.59	1.16	2.24	1.42	2.12	1.40	1.6	0.206	0.012	1.2	0.278	0.009	0.9	0.353	0.006
Have you worried about other people seeing rolls of fat around your waist or stomach?	2.33	1.88	1.77	1.16	3.25	1.89	2.31	1.16	8.7	0.004	0.060	7.1	0.008	0.050	0.4	0.676	0.001
Have you felt that it is not fair that other people are thinner than you?	1.54	1.44	1.10	0.38	1.98	1.50	1.81	0.98	2.9	0.092	0.021	7.7	0.006	0.054	0.7	0.421	0.005
Has seeing your reflection (e.g. in a mirror or shop window) made you feel bad about your shape?	2.17	1.88	1.38	0.85	2.82	1.72	1.92	1.06	11.0	0.001	0.075	5.7	0.019	0.040	0.0	0.945	0.000
Have you been particularly self-conscious about your shape when in the company of other people?	4.33	1.81	3.74	1.25	4.57	1.17	4.00	1.13	6.9	0.010	0.049	1.2	0.269	0.009	0.1	0.823	0.000
Has worry about your shape made you feel you ought to exercise?	2.54	1.47	3.38	1.63	3.00	1.22	3.58	1.47	6.0	0.016	0.042	1.7	0.198	0.012	0.2	0.651	0.002
**Total score**	80.14	32.97	71.86	19.45	101.19	35.29	88.74	25.61	6.6	0.011	0.47	14.3	0.000	0.96	0.0	0.847	0.000
